# Truncated vitronectin with E-cadherin enables the xeno-free derivation of human embryonic stem cells

**DOI:** 10.1038/s41598-023-42236-5

**Published:** 2023-09-12

**Authors:** Tereza Souralova, Daniela Hulinova, Michal Jeseta, Pavel Ventruba, Ales Hampl, Irena Koutna

**Affiliations:** 1 Department of Histology and Embryology, Faculty of Medicine, Masaryk University, Kamenice 5, 625 00 Brno, Czech Republic; 2 International Clinical Research Center, Cell and Tissue Engineering Facility, St. Anne’s University Hospital, Pekarska 53, 602 00 Brno, Czech Republic; 3 Department of Experimental Biology, Faculty of Science, Masaryk University, Kamenice 5, 625 00 Brno, Czech Republic; 4 Department of Gynecology and Obstetrics, Faculty of Medicine, Center of Assisted Reproduction, Masaryk University Brno and University Hospital, Obilni Trh 11, 602 00 Brno, Czech Republic; 5 International Clinical Research Center, Cell and Tissue Regeneration, St. Anne’s University Hospital, Pekarska 53, 602 00 Brno, Czech Republic

**Keywords:** Embryonic stem cells, Stem cells, Pluripotent stem cells, Embryonic stem cells, Translational research, Stem-cell research, Preclinical research

## Abstract

Human embryonic stem cells (hESCs) have unique abilities that enable their use in cell therapy, disease modeling, and drug development. Their derivation is usually performed using a feeder layer, which is undefined and can potentially cause a contamination by xeno components, therefore there is a tendency to replace feeders with xeno-free defined substrates in recent years. Three hESC lines were successfully derived on the vitronectin with a truncated N-terminus (VTN-N) in combination with E-cadherin in xeno-free conditions for the first time, and their undifferentiated state, hESC morphology, and standard karyotypes together with their potential to differentiate into three germ layers were confirmed. These results support the conclusion that the VTN-N/E-cadherin is a suitable substrate for the xeno-free derivation of hESCs and can be used for the derivation of hESCs according to good manufacturing practices.

## Introduction

Human embryonic stem cells (hESCs) proliferate almost indefinitely without the loss of pluripotency, the potential to differentiate into any cell type in a human body^1^. Self-renewal and pluripotency allow to produce high number of hESCs and subsequently hESC-derived desired cell types that can be used in regenerative therapies, drug development, and disease modeling^2–4^.

Regardless of the intended use of hESCs or hESC-derived cells, consistent and reproducible results are desired. One factor that contributes to inconsistent results is the variability of the derivation and subsequent culture conditions. The allogeneic or xenogeneic origin of some feeders and substrates, e.g., Matrigel, mouse embryonic fibroblasts, or human foreskin fibroblasts, can be potentially the source of variability and moreover viral or bacterial contamination^5–7^.

The high variability of feeders and some substrates can be lowered using chemically defined recombinant xeno-free substrates. Despite the fact that defined recombinant xeno-free substrates as vitronectin, laminin 521, collagen IV, or fibronectin were used for the hESC culture^8–12^, only laminin 521^5^ and recombinant laminin-511 E8 protein fragments^13^ were used for the hESC derivation.

Vitronectin is a glycoprotein found in the extracellular matrix and blood that promotes cell adhesion and spreading^15,16^. Its truncated form VTN-N is a defined, recombinant human protein that is used for human pluripotent stem cell culture and differentiation^12,16–18^. Despite its pluripotency-maintaining properties, any form of vitronectin has never been used for the derivation of hESCs according to current literature.

In this article, we describe for the first time the derivation of three hESC lines on VTN-N with attachment support provided by E-cadherin and compare their properties with the hESC lines derived on laminin 521 according to GMP that were established by our group previously^19^.

## Methods

### Donor testing

Embryo donors were tested for the presence of HIV1/2, hepatitis B, hepatitis C, and syphilis with negative results as previously described^19^. Testing was performed according to procedures of the CAR University Hospital Brno embryological laboratory (Brno, Czech Republic).

### Embryo preparation and transport

Embryos were thawed using Warm Cleave or Warm Blast media (Vitrolife, Västra Frölunda, Sweden) at the CAR University Hospital Brno embryological laboratory (Brno, Czech Republic), a day or two days (according to frozen stages) before the transport as previously described^19^. For lower embryo stages, the cultivation to the blastocyst stage in Blastocyst Medium (cat. no. G20722, Cook Medical, Bloomington, USA) was performed. After reaching the blastocyst stage, the disruption of the zona pellucida was executed using a laser (OCTAX NaviLase). Prepared embryos in the hatched blastocyst stage were transferred to the Sydney IVF gamete buffer medium (cat. no. G48258, Cook Medical, Bloomington, USA) and transported using a temperature-controlled transport incubator at 37 °C (portable incubator, Minitube).

### Derivation

The embryo derivation procedure was initiated immediately upon receipt of the embryos, following established protocols^19^. Each individual embryo was carefully introduced into a well of a 4-well dish (Thermofisher Scientific, San Jose, CA, USA) filled with pre-warmed Sydney IVF gamete buffer medium, situated on a stereomicroscope plate maintained at 37 °C. Subsequently, the embryo was delicately transferred into a small droplet of Sydney IVF gamete buffer medium (cat. no. G48258, Cook Medical, Bloomington, USA), overlaid with Sydney IVF culture oil (cat. no. G44990, Cook Medical, Bloomington, USA), using a denuding micropipette (cat. 005-300-A, Microtech IVF, Czech Republic). Throughout the process, each embryo was handled separately, employing biopsy micropipettes (cat. 004-35-30A, Microtech IVF, Czech Republic) and holding micropipettes (cat. 001-120-30H, Microtech IVF, Czech Republic). The inner cell mass (ICM) was aspirated using a biopsy micropipette and carefully positioned in parallel with a holding micropipette, slightly overlapping to enable efficient mechanical biopsy using a rapid swinging motion (previously published video: https://www.mdpi.com/1422-0067/23/20/12500#B41-ijms-23-12500). Subsequently, the denuding micropipette (cat. 005-150-C, Microtech IVF, Czech Republic) was employed to manipulate the ICM, which was then transferred into an individual well of a 4-well dish pre-coated with 10.0 µg/mL (1.6 µg/cm^2^) of recombinant VTN-N (cat. no. A14700, ThermoFisher Scientific), and 2.2 µg/mL (0.34 µg/cm^2^) of E-cadherin (R&D Systems) diluted in phosphate-buffered saline (PBS). Coating of 4-well dishes with VTN-N/E-cadherin was performed for 1 h at room temperature. Throughout the entire derivation process, the microscope plates were maintained at a constant temperature of 37 °C. E-cadherin was added only for the derivation until the first passage. The derivation medium consisted of NutriStem® hPSC XF Medium (Biological Industries, Beit-Haemek, Israel), supplemented with 20 mg/mL of human serum albumin (Vitrolife), and 10 µM of the ROCK inhibitor (Y27632, GMP, Bio-techne). The derivation medium was used only for the first three days of the derivation, then the NutriStem® hPSC XF Medium was changed daily.

### Culture conditions

The derived hESCs were cultured under hypoxic culture conditions (5% O_2_, 5% CO_2_, 37 °C) for the first three passages, then under normoxic conditions (5% CO_2_, 37 °C) in a NutriStem® hPSC XF Medium (Biological Industries) with a daily medium change as previously described^19^. The cells were passaged mechanically by an insulin syringe (B.Braun) and cultured on 5.0 µg/mL (0.6 µg/cm^2^) recombinant VTN-N (cat. no. A14700, ThermoFisher Scientific) for the first three passages^17^. From passage four, non-enzymatic subculturing utilizing 0.5 mM EDTA was conducted once the cell population achieved 70% confluence. To promote single cell survival and facilitate the passaging process, a ROCK inhibitor (Y27632, GMP, Bio-techne) at a concentration of 10 µM was applied, both 1 h before and immediately after the passage for the following 24 h. Following the one-hour treatment with the ROCK inhibitor, the cells were gently washed with phosphate-buffered saline (PBS, Gibco), dissociated into clumps using 0.5 mM EDTA (Invitrogen), and subsequently transferred onto culture surfaces coated with 5.0 µg/mL (0.6 µg/cm^2^) recombinant VTN-N (cat. no. A14700, ThermoFisher Scientific).

### Determination of growth curve and population doubling time

Cells were cultured in 24-well plates at a seeding density of 2 × 10^4^ cells per well and harvested between days 1 and 6 post-seeding. The viability of cells was assessed using the Countess III Automated Cell Counter (Thermo Fisher Scientific) with trypan blue exclusion. A growth curve was constructed to depict the cell population dynamics over time. The proliferation rate, expressed as Population Doubling Time (PDT), was determined at the inflection point of the growth curve. The PDT was calculated using the formula PDT = T ln(2)/ln(A/A0), where T represents the cultivation time in hours, A is the final cell number, A0 corresponds to the initial cell number and ln is natural logarithm^20^.

### Flow cytometry

Cells were washed in phosphate-buffered saline (PBS, Gibco), resuspended in a PBS/EDTA (Invitrogen)/bovine serum albumin (BSA, Pan Biotech) solution, and incubated with antibodies for 10 min at 4 °C, after which the cells were rinsed with PBS, spun for 4 min/300 g, resuspended in PBS, and analyzed by a BD FACS Canto II device (BD Biosciences) using FACSDiva (BD Biosciences) and Flowing Software (The University of Turku) as previously described^19^. The antibodies used were: human anti-TRA-1-60-PE, 1:75 (cat: 130-122-921, Miltenyi Biotec), human anti-SSEA-4-PE, 1:150 (cat: 130-122-914, Miltenyi Biotec), and human anti-TRA-1-81-APC, 1:90 (cat: 17-8883-42, Thermo Fisher Scientific, San Jose, CA, USA).

### EB formation and cell differentiation

Differentiation to the three germ layers was supported by the medium: DMEM/F12 (Gibco), 15% knockout-serum replacement (Gibco), 1% non-essential amino acids (Sigma), 1% Glutamax (Gibco), 1% ZellShield (Minerva Biolabs), and 0.2% 2-Mercaptoethanol (Gibco) as previously described^19^. The cells were passed into a low attachment 96-well dish (20 × 10^3^ cells/well) and cultured for 1 week until embryoid bodies were formed. The embryoid bodies were then transferred to 4-well dishes coated with 5.0 µg/mL (0.6 µg/cm^2^) recombinant VTN-N (cat. no. A14700, ThermoFisher Scientific), where they were allowed to attach and culture for another 14 days.

### Immunocytochemistry

The cells were fixed using cold 4% paraformaldehyde (Sigma) for a duration of 20 min, followed by permeabilization using 0.2% Triton × (Sigma) for 30 min. Subsequently, a blocking step was performed for 1 h in a solution of 2.5% Bovine Serum Albumin (BSA, Pan Biotech) in phosphate-buffered saline (PBS, Gibco), supplemented with 0.1% Tween 20 (Sigma), as previously described^19^. For immunostaining, the fixed cells were exposed to primary antibodies overnight at 4 °C. The primary antibodies used were as follows: mouse anti-OCT3/4 at 1:200 dilution (cat: sc-5279, Santa Cruz Biotechnology, Dallas, TX, USA), rabbit anti-NANOG at 1:200 dilution (cat: 4903, Cell Signaling Technology), mouse anti-SOX2 at 1:100 dilution (cat: MAB2018, R&D Systems), mouse anti-α-actin at 1:200 dilution (cat: sc-130616, Santa Cruz Biotechnology), goat anti-FOXA2 at 1:200 dilution (cat: AF2400, R&D Systems), mouse anti-β3 Tubulin at 1:200 dilution (cat: sc-850005, Santa Cruz Biotechnology), goat anti-PDX1 at 1:17 dilution (cat: AF2419, R&D Systems), rabbit anti-brachyury at 1:200 dilution (cat: sc-20109, Santa Cruz Biotechnology), and mouse anti-OTX2 at 1:200 dilution (cat: sc-514195, Santa Cruz Biotechnology). Subsequently, secondary antibodies were applied for 1 h, using the following dilutions: anti-mouse Alexa 555 at 1:500 (cat: 4409, Cell Signaling Technology), anti-rabbit Alexa 488 at 1:500 (cat: 4412, Cell Signaling Technology, Danvers, MA, USA), and donkey anti-goat NL557 at 1:500 (cat: 4412, Cell Signaling). To visualize cell nuclei, the stained cells were treated with 1 µg/mL of DAPI (4′,6-diamidino-2-phenylindole) and subsequently observed under a fluorescence microscope. Image acquisition was performed using Acquiarium software (v2012-06-12, Faculty of Informatics, Masaryk University).

### Karyotyping

Cells were mitotically arrested by adding 0.4 μg/mL KaryoMAX™ Colcemid™ Solution (Thermofisher) and subsequently incubated for 2 h under standard culture conditions (5% CO_2_, 37 °C) as previously described^19^. Following the detachment of cells with TrypLe express and a 25 min treatment with the hypotonic solution (DMEM/F12 with demineralized water in ratio 1:3), the cells were fixed with 4 °C methanol and acetic acid (3:1). A karyotype analysis was performed by the Cytogenetic Laboratory Brno (Brno, Czech Republic) with Giemsa-banding and microscopic examination. At least 40 metaphase spreads/samples were analyzed at a resolution of 450–500 bands/haploid set.

### Ethics approval and consent to participate

The study was approved by the Ethics Board of the Faculty of Medicine of Masaryk University (name of the project: Clinical grade human embryonic stem cells—derivation and characterization, approval number: 16/2017, date of approval: 26/06/2017). All research was performed in accordance with relevant regulations. Informed consent was obtained from all embryo donors involved in the project.

## Results

### VTN-N/E-cadherin is a suitable surface for the derivation of human embryonic stem cells

Three hESC lines, MUES 10, MUES 11, and MUES 12, were established with the use of VTN-N/E-cadherin from 20 embryos in total (see Table 1). The rate of hESC line derivation is 15.0%. Three individual derivations were performed with 3–9 embryos per derivation.

**Table 1:** Overview of the derivations of hESC lines derived on VTN-N/E-cadherin.

Derivation no	Number of embryos^a^	hESC lines
1	9	0
2	8	2
3	3	1
	20	3

^a^All transported embryos are involved even though some of them started to disintegrate after the thaw/transport.

**Figure 1:**
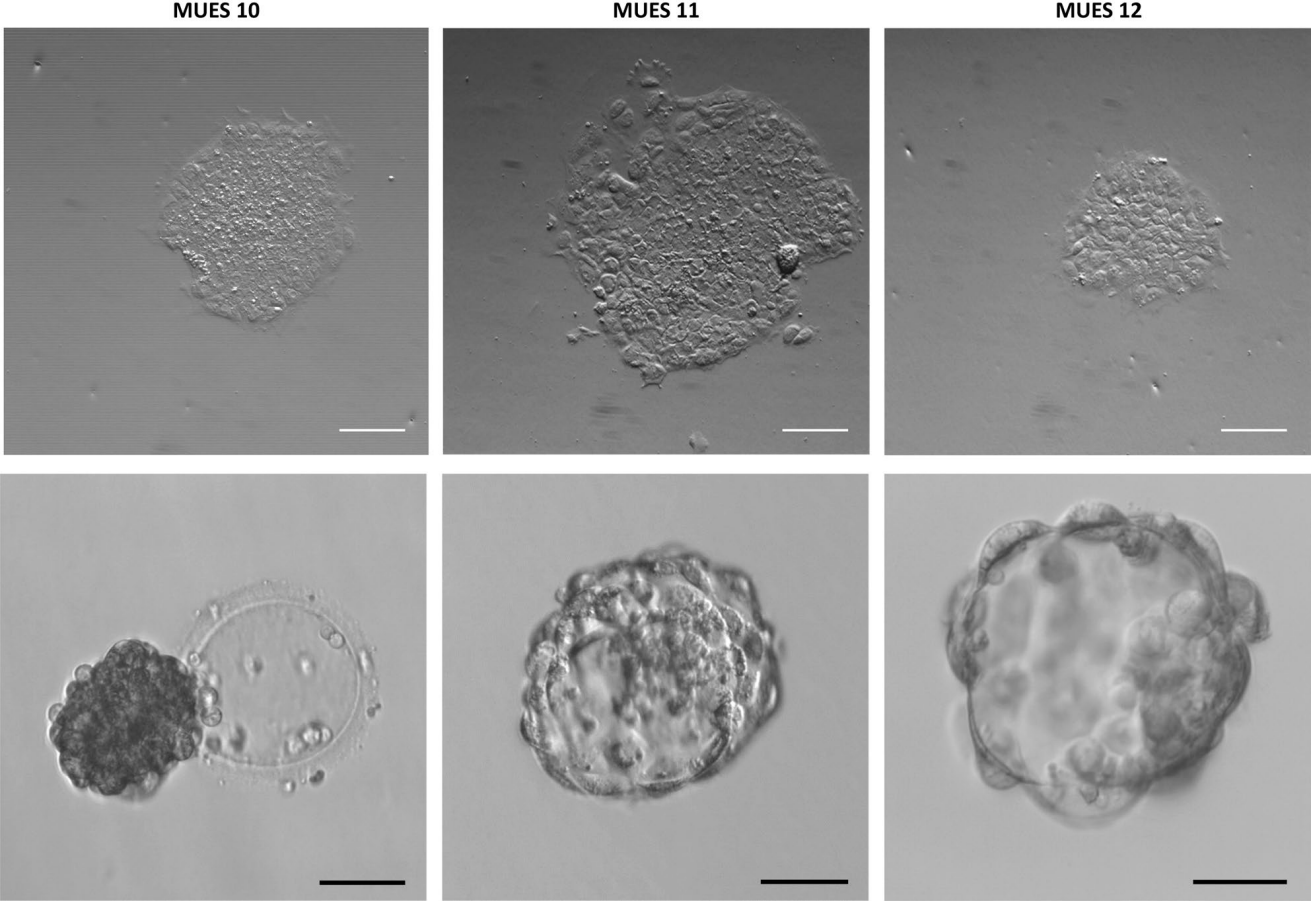
Colonies in early passages for MUES 10 (p1), MUES 11 (outgrowth) and MUES 12 (p1) hESC lines (scale bar = 10 µm) and photos of blastocysts prior the derivation for hESC lines MUES 10, MUES 11, and MUES12 (scale bar = 50 µm).

Fully-hatched blastocysts with visible inner cell masses (see Fig. 1) were used for the derivation of hESC lines MUES 11 and MUES 12. The collapsed hatching blastocyst (see Fig. 1) was used for the derivation of MUES 10. The separation of inner cell mass was impossible due to its collapsed state therefore the blastocyst was split into several clumps. Inner cell masses and clumps were transferred on 10.0 µg/mL (1.6 µg/cm^2^) VTN-N enriched with 2.2 µg/mL (0.34 µg/cm^2^) E-cadherin. The differentiation was observed in the first passage only in MUES 11 (see Fig. 1) although the differentiation occurred in all three hESC lines in early passages until the hESC phenotype was stabilized (data not shown).

**Figure 2:**
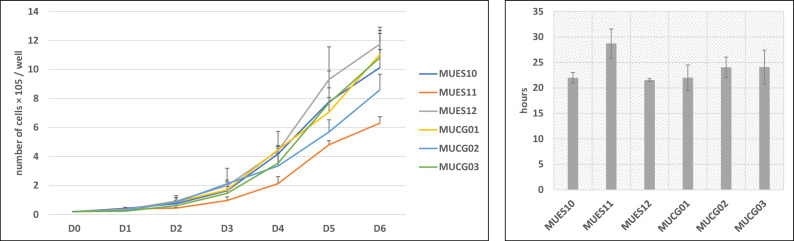
Growth curve (**A**) was established and population doubling time (**B**) calculated in hESC lines MUES 10, MUES 11, MUES 12, MUCG01, MUCG02, and MUCG03. Results refer to the mean ± SD, n = 3.

The growth curve was established and population doubling time was calculated in hESC lines MUES 10 (p18–p22), MUES 11 (p18–p21), and MUES 12 (p21–p25) (derived on VTN-N/E-cadherin) that were compared to MUCG01 (p23–p32), MUCG02 (p24–p28), MUCG03 (p34–p38) that were derived on laminin 521/E-cadherin. There was no significant difference in population doubling time (MUES 10 = 22.0 ± 1.0, MUES 11 = 28.7 ± 2.9, MUES 12 = 21.6 ± 0.3, MUCG01 = 22.0 ± 2.5, MUCG02 = 24.1 ± 2.0, MUCG03 = 24.1 ± 3.3 h) among hESC lines derived on VTN-N/E-cadherin and hESC lines derived on laminin 521/E-cadherin (see Fig. 2).

### hESC lines derived on VTN-N/E-cadherin maintain undifferentiated state, hESC morphology and standard karyotypes

**Figure 3:**
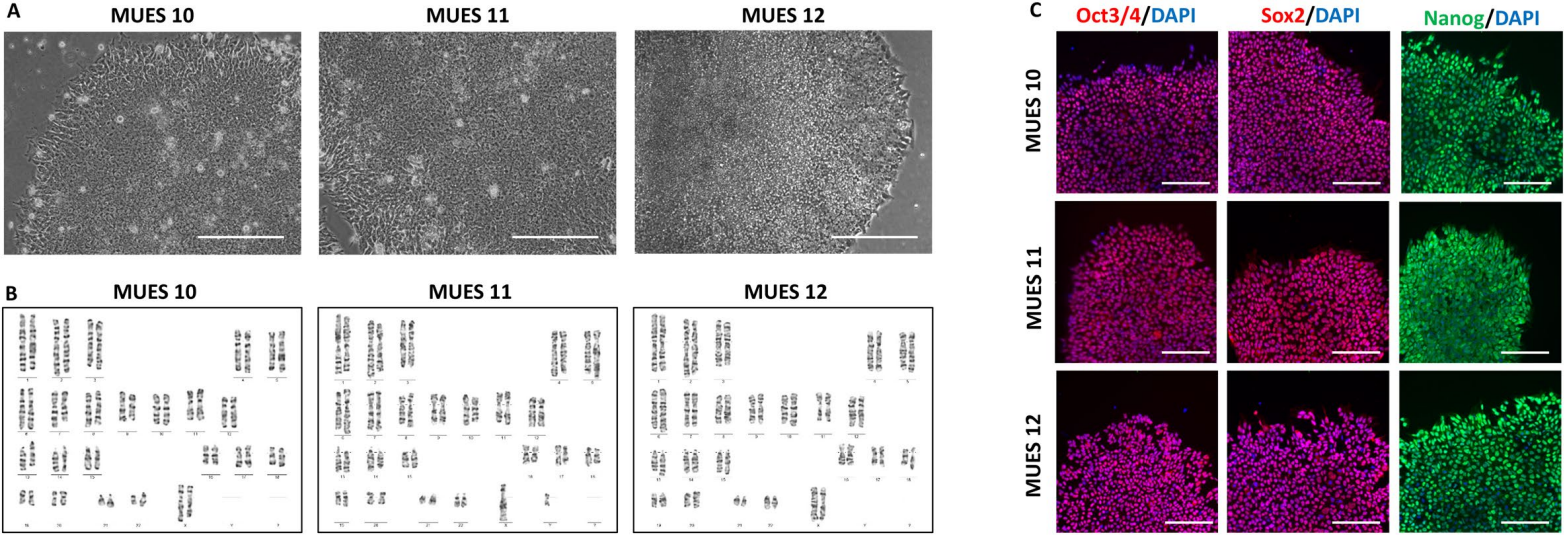
hESC lines MUES 10 (XX), MUES 11 (XY), and MUES 12 (XX) have characteristic hESC morphology; scale bar = 50 µm (**A**) and karyotypes (**B**). hESC lines MUES 10, MUES 11, and MUES 12 are positive for markers of undifferentiated state OCT3/4, SOX2, and NANOG; scale bar = 100 µm (**C**).

Our established cell lines MUES 10 (p20), MUES 11 (p19), and MUES 12 (p22) have the characteristic hESC morphologies, i.e., colonies are flat with bright edges, the cells in the middle of the colony are small and round with large nucleus and visible nucleoli. The cells on the edges are slightly bigger (see Fig. 3A).

Karyotyping was performed in p20 for MUES 10, in p19 for MUES 11, and in p19 for MUES 12. All three lines have standard karyotypes, MUES 10 (XX), MUES 11 (XY), and MUES 12 (XX) (see Fig. 3B).

hESC lines MUES 10 (p12), MUES 11 (p8), and MUES 12 (p9) are positive for OCT3/4, SOX2, and NANOG markers of undifferentiated state measured by immunocytochemistry (see Fig. 3C) and > 90% of cells for MUES 10 (p16, p20), MUES 11 (p16, p19), MUES 12 (p20, p22), MUCG01 (p26, p30) MUCG02 (p24, p26), and MUCG03 (p32, p36) are positive for TRA-1-60, TRA-1-81, and SSEA4 markers of undifferentiated state measured by flow cytometry (see Table 2). These results are comparable to three hESC lines MUCG01, MUCG02, and MUCG03 derived on laminin 521 as these lines are positive for OCT3/4, SOX2, and NANOG markers of undifferentiated state measured by immunocytochemistry (data not shown) and > 90% of cells are positive for TRA-1-60, TRA-1-81, and SSEA4 markers of undifferentiated state (see Table 2).

**Table 2:** Markers of undifferentiated state TRA-1-60, TRA-1-81, and SSEA4 measured in hESC lines MUES 10, MUES 11, MUES 12, MUCG01, MUCG02, and MUCG03.


hESC line	TRA-1-60^+^ (%)	TRA-1-81^+^ (%)	SSEA4^+^ (%)
MUES 10	97.12 ± 1.64	95.33 ± 0.98	99.84 ± 0.04
MUES 11	96.33 ± 3.13	91.95 ± 1.81	99.93 ± 0.05
MUES 12	99.43 ± 0.45	98.40 ± 1.03	99.75 ± 0.16
MUCG01	99.75 ± 0.07	97.56 ± 1.59	99.03 ± 0.91
MUCG02	99.63 ± 0.17	98.97 ± 0.13	99.46 ± 0.09
MUCG03	99.77 ± 0.09	98.20 ± 0.87	99.82 ± 0.03

Results refer to the mean ± SD, n = 2.

All three hESC lines MUES 10 (MUNIe010-A), MUES 11 (MUNIe011-A), and MUES 12 (MUNIe012-A), were registered at hPSCreg (https://hpscreg.eu/).

### hESC lines derived on VTN-N/E-cadherin differentiate into all three germ layers

**Figure 4:**
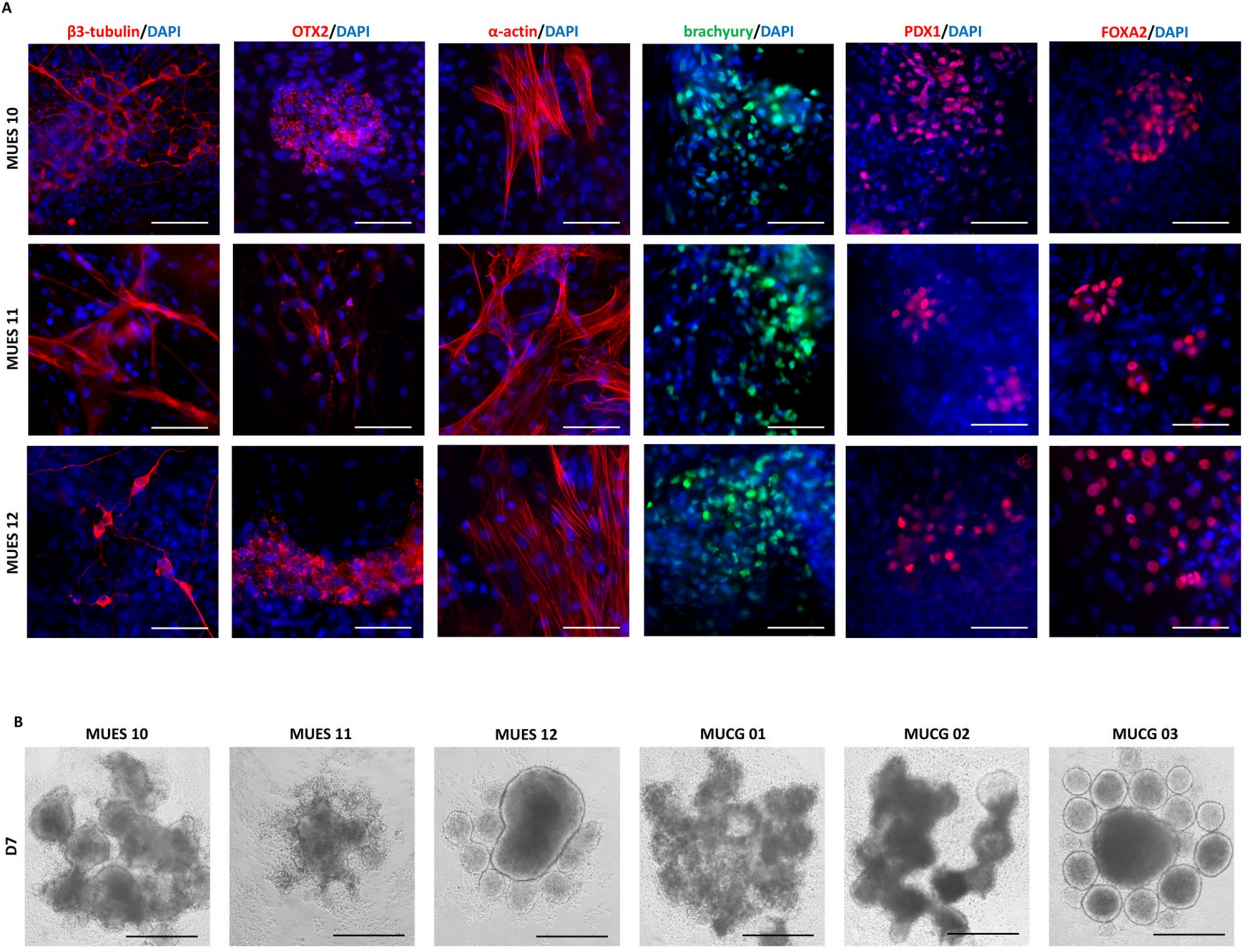
Ectoderm markers (β3-tubulin, OTX2), mesoderm markers (α-actin, brachyury), and endoderm markers (PDX1, FOXA2) were detected after differentiation of hESC lines MUES 10, MUES 11, and MUES 12; scale bar = 50 µm (**A**). Embryoid bodies formation in D7 (representative photos; n = 3) for hESC lines MUES 10, MUES 11, MUES 12 derived on VTN-N/E-cadherin and MUCG01, MUCG02, and MUCG03 derived on laminin 521/E-cadherin; scale bar = 50 µm (**B**).

hESC lines MUES 10 (p12), MUES 11 (p13), and MUES 12 (p15) differentiate into all three germ layers. The lines are positive for ectoderm markers (β3-tubulin, OTX2), mesoderm markers (α-actin, brachyury), and endoderm markers (PDX1, FOXA2) detected by immunocytochemistry (see Fig. 4A).

There is variability in size, shape, and number of EBs formed from hESC lines MUES 10 (p16, p17, p18), MUES 11 (p16, p17, p18), and MUES12 (p19, p20, p21) after 7-day culture (see Fig. 4B). The comparable variability was observed in EBs formed from hESC lines MUCG01 (p26, p27, p28), MUCG02 (p24, p24, p24; separate repetitions), and MUCG03 (p32, p33, p34), that were derived on laminin 521. There was observed a decrease in markers of the undifferentiated state TRA-1-60, TRA-1-81, and SSEA4 in 7-day EBs (see Table 3).

**Table 3:** Markers of undifferentiated state TRA-1-60, TRA-1-81, and SSEA4 measured in 7-day EBs formed with the use of hESC lines MUES 10, MUES 11, MUES 12 derived on VTN-N/E-cadherin and MUCG01, MUCG02, and MUCG03 derived on laminin 521/E-cadherin.


7-Day EBs from hESC line	TRA-1-60^+^ (%)	TRA-1-81^+^ (%)	SSEA4^+^ (%)
MUES 10	58.75 ± 8.35	42.47 ± 4.19	83.37 ± 4.63
MUES 11	64.61 ± 8.90	34.86 ± 6.55	87.54 ± 1.40
MUES 12	57.68 ± 21.64	56.85 ± 9.19	79.48 ± 8.17
MUCG01	85.17 ± 4.31	53.84 ± 5.09	84.20 ± 7.57
MUCG02	85.56 ± 9.32	68.64 ± 19.52	91.17 ± 2.67
MUCG03	94.58 ± 1.50	85.47 ± 3.18	86.62 ± 4.25

Results refer to the mean ± SD, n = 3.

## Discussion

The derivation of hESC lines on VTN-N/E-cadherin was performed with the use of hatching or fully hatched blastocysts that were thawed at center of assisted reproduction. The zona pellucida was disrupted by laser followed by transport to our laboratory. We hypothesize that the disruption of zona pellucida may cause the degradation of blastocyst during transport, therefore it would be beneficial to collect and analyze data regarding this issue. However, we achieved a 15.0% derivation success rate with the use of 20 blastocysts in total. It is difficult to compare the success rate as no group has derived new hESC lines on VTN-N/E-cadherin yet, but we used laminin 521/E-cadherin in our previous setting and achieved a 7.9% success rate with the use of 38 embryos in total^19^. Takada et al. derived 1 clinical-grade hESC line on recombinant laminin-511 E8 protein fragments in period one with 7.1% efficiency (14 blastocysts, 1 hESC line) which increased to 45.5% in period two (11 blastocysts, 5 hESC lines)^13^. Despite the lack of statistically sufficient amount of data, VTN-N/E-cadherin has almost two times better derivation success rate compared to derivation of new lines on laminin 521/E-cadherin by our group^19^.

Researchers in the laboratory of James Thomson found that the VTN-N supports hESC attachment and survival better than wild-type vitronectin when used in conjunction with Essential 8™ Medium^17^. Therefore we decided to use the VTN-N for the derivation instead of its wild-type form. Moreover, its supporting properties are complemented by its availability in GMP quality, so it can be potentially used in the GMP manufacture of hESCs and hESC-derived cells used in cell therapy^21,22^. Moreover, GMP VTN-N is a chemically defined xeno-free substrate that is easy to use for coating and is more cost-effective in comparison to GMP laminin 521.

The E-cadherin supports the survival, self-renewal, and pluripotent state of isolated hESCs^5,14^. We decided to double the VTN-N concentration usually used for the cell culture and add E-cadherin to support the survival of isolated inner cell mass and increase the establishment efficiency. We designed this condition based on our previous experience with the derivation of hESCs on laminin 521/E-cadherin according to GMP.

We observed characteristic hESC morphology in all three hESC lines derived on VTN-N/E-cadherin^23^. Unfortunately, it was impossible to make photos of outgrowths for MUES 10 and MUES 11 as they were on the edge of the well. We observed the occurrence of outgrowths more often on the edges of the wells in our previous derivations on laminin 521 too (data not shown).

We observed the variability among various hESC lines, derived either on laminin 521/E-cadherin or VTN-N/E-cadherin, during the embryoid bodies’ development. It is difficult to make clear conclusions as three hESC lines were used, therefore there is not enough data for statistical analysis. However, it is possible these differences are caused by the biological variability of hESC lines and are independent of the derivation and culture surface^24^. Although the same number of cells was used at the beginning of the experiment, it was impossible to infer how many cells will be involved in the creation of one single EB, therefore it would be helpful to optimize the creation of EBs to make results more comparable^25^. It would be beneficial to collect more data focusing on undifferentiated state and differentiation of more hESC lines derived on VTN-N/E-cadherin and find a statistically supported conclusion if a VTN-N/E-cadherin is a superior surface for hESC derivation.

## Conclusions

We used VTN-N/E-cadherin for the derivation of hESCs for the first time, derived three hESC lines, and confirmed their undifferentiated state, characteristic hESC morphology, and standard karyotypes as well as their potential to differentiate into three germ layers. All three hESC lines derived on VTN-N/E-cadherin form EBs with similar variability as hESC lines derived on laminin 521/E-cadherin. These results support the conclusion that the VTN-N in combination with E-cadherin is a suitable substrate for the derivation of hESCs.

## Data Availability

All three hESC lines MUES 10 (MUNIe010-A), MUES 11 (MUNIe011-A), and MUES 12 (MUNIe012-A), were registered at hPSCreg (https://hpscreg.eu/). The datasets used and/or analyzed during the current study available from the corresponding author on reasonable request.

## References

[1] Thomson JA (1998). Embryonic stem cell lines derived from human blastocysts. Science.

[2] Eguizabal C, Aran B, Chuva de Sousa Lopes SM, Geens M, Heindryckx B, Panula S (2019). Two decades of embryonic stem cells: A historical overview. Hum. Reprod. Open.

[3] Lin X, Tang J, Lou Y-R (2021). Human pluripotent stem-cell-derived models as a missing link in drug discovery and development. Pharmaceuticals.

[4] Yamanaka S (2020). Pluripotent stem cell-based cell therapy—Promise and challenges. Cell Stem Cell.

[5] Rodin S, Antonsson L, Niaudet C, Simonson OE, Salmela E, Hansson EM (2014). Clonal culturing of human embryonic stem cells on laminin-521/E-cadherin matrix in defined and xeno-free environment. Nat. Commun..

[6] Marchini A, Gelain F (2022). Synthetic scaffolds for 3D cell cultures and organoids: Applications in regenerative medicine. Crit. Rev. Biotechnol..

[7] Aisenbrey EA, Murphy WL (2020). Synthetic alternatives to Matrigel. Nat. Rev. Mater..

[8] Albalushi H, Kurek M, Karlsson L, Landreh L, Kjartansdóttir KR, Söder O (2018). Laminin 521 stabilizes the pluripotency expression pattern of human embryonic stem cells initially derived on feeder cells. Stem Cells Int..

[9] Braam SR, Zeinstra L, Litjens S, Ward-van Oostwaard D, van den Brink S, van Laake L (2008). Recombinant vitronectin is a functionally defined substrate that supports human embryonic stem cell self-renewal via αVβ5 integrin. Stem Cells.

[10] Xu C, Inokuma MS, Denham J, Golds K, Kundu P, Gold JD (2001). Feeder-free growth of undifferentiated human embryonic stem cells. Nat. Biotechnol..

[11] Fan Y, Wu J, Ashok P, Hsiung M, Tzanakakis ES (2015). Production of human pluripotent stem cell therapeutics under defined xeno-free conditions: Progress and challenges. Stem Cell Rev. Rep..

[12] Prowse ABJ, Doran MR, Cooper-White JJ, Chong F, Munro TP, Fitzpatrick J (2010). Long term culture of human embryonic stem cells on recombinant vitronectin in ascorbate free media. Biomaterials.

[13] Takada K, Nakatani R, Moribe E, Yamazaki-Fujigaki S, Fujii M, Furuta M (2022). Efficient derivation and banking of clinical-grade human embryonic stem cell lines in accordance with Japanese regulations. Regener. Therapy.

[14] Li L, Bennett SAL, Wang L (2012). Role of E-cadherin and other cell adhesion molecules in survival and differentiation of human pluripotent stem cells. Cell Adhes. Migr..

[15] Schvartz I, Seger D, Shaltiel S (1999). Vitronectin. Int. J. Biochem. Cell Biol..

[16] Hayashi Y, Furue MK (2016). Biological effects of culture substrates on human pluripotent stem cells. Stem Cells Int..

[17] Chen G, Gulbranson DR, Hou Z, Bolin JM, Ruotti V, Probasco MD (2011). Chemically defined conditions for human iPS cell derivation and culture. Nat. Methods..

[18] Gil J-E, Woo D-H, Shim J-H, Kim S-E, You H-J, Park S-H (2009). Vitronectin promotes oligodendrocyte differentiation during neurogenesis of human embryonic stem cells. FEBS Lett..

[19] Souralová T, Řeháková D, Ješeta M, Tesařová L, Beránek J, Ventruba P (2022). The manufacture of xeno- and feeder-free clinical-grade human embryonic stem cell lines: First step for cell therapy. Int. J. Mol. Sci..

[20] Tesarova L, Jaresova K, Simara P, Koutna I (2020). Umbilical cord-derived mesenchymal stem cells are able to use bFGF treatment and represent a superb tool for immunosuppressive clinical applications. Int. J. Mol. Sci..

[21] Zhu X-Y, Chen Y-H, Zhang T, Liu S-J, Bai X-Y, Huang X-Y (2021). Improvement of human embryonic stem cell-derived retinal pigment epithelium cell adhesion, maturation, and function through coating with truncated recombinant human vitronectin. Int. J. Ophthalmol..

[22] da Cruz L, Fynes K, Georgiadis O, Kerby J, Luo YH, Ahmado A (2018). Phase 1 clinical study of an embryonic stem cell-derived retinal pigment epithelium patch in age-related macular degeneration. Nat. Biotechnol..

[23] Orozco-Fuentes S, Neganova I, Wadkin LE, Baggaley AW, Barrio RA, Lako M (2019). Quantification of the morphological characteristics of hESC colonies. Sci. Rep..

[24] Odorico JS, Kaufman DS, Thomson JA (2001). Multilineage differentiation from human embryonic stem cell lines. Stem Cells.

[25] Moon S-H, Ju J, Park S-J, Bae D, Chung H-M, Lee S-H (2014). Optimizing human embryonic stem cells differentiation efficiency by screening size-tunable homogenous embryoid bodies. Biomaterials.

